# Knowledge, Attitude, Practice, and Associated Factors of Postoperative Pain Management Among Nurses

**DOI:** 10.1155/prm/6653069

**Published:** 2025-08-01

**Authors:** Bereket Samuel, Man Ye

**Affiliations:** ^1^Clinical Nursing Teaching and Research Section, The Second Xiangya Hospital of Central South University, Changsha, Hunan, China; ^2^Xiangya Nursing School of Central South University, Changsha, Hunan, China

**Keywords:** attitude, knowledge, postoperative pain management, practice

## Abstract

**Background:** Nurses play a crucial role in pain management through adherence to protocols, accurate pain assessment, and personalized pain relief strategies. However, a gap exists between nurses' ability to perceive pain and patients' actual needs. In Ethiopia, postoperative pain management practices are inadequate, and there is limited research on nurses' pain cognition.

**Aim:** To evaluate the knowledge, attitudes, practices, and associated factors regarding postoperative pain management among nurses at Wolaita Sodo Comprehensive Specialized Hospital in Ethiopia.

**Methods:** A cross-sectional study design involving 124 nurses was utilized. Data were collected using the Knowledge Attitude Survey regarding pain and the Nurses Carrying Behaviors Checklist. Statistical analysis was conducted using SPSS Version 28, employing descriptive statistics, one-way ANOVA, independent sample *t*-tests, and Pearson correlation coefficients. Multiple linear regressions were used to identify factors associated with pain management practices, with statistical significance set at a *p* value below 0.05.

**Results:** The mean knowledge, attitude, and practice scores were 49.51 ± 9.51, 43.04 ± 14.72, and 71.05 ± 10.53, respectively. Positive correlations were found between knowledge and practices (*r* = 0.348, *p* < 0.001) and between attitudes and practices (*r* = 0.247, *p*=0.006). Training on pain, pain experience, work experience, and marital status were independent influencing factors for practice toward postoperative pain management.

**Conclusion:** The study highlights critical gaps in nurses' knowledge and practices regarding postoperative pain management, particularly in opioid safety, dose conversion, and withdrawal symptoms. Over half of the nurses had inadequate knowledge, and most exhibited poor practices. Although negative attitudes were prevalent, training, experience, and personal pain exposure contributed to improved practices. Enhancing structured education, clinical mentoring, and institutional support is essential to improve postoperative pain care.

## 1. Introduction

The International Association for the Study of Pain defines pain as an unpleasant sensory and emotional experience associated with, or resembling, actual or potential tissue damage [1]. This definition emphasizes that pain affects individuals not only physically but also psychologically and emotionally. Postoperative pain, a form of acute pain arising from surgical trauma and inflammation, can lead to nerve injury [2].

Globally, an estimated 312.9 million surgeries occur annually, with a surgical rate of 4469 per 100,000 people [3]. Despite advancements in perioperative interventions and management strategies, a significant proportion of surgical patients still experience substantial acute postoperative pain [4, 5]. Pain remains one of the most common reasons for seeking healthcare and hospital admission, accounting for nearly two-thirds of emergency department visits in the United States [6].

In a study conducted in India, Sommer et al. reported that 41% of 1490 postoperative patients experienced moderate to severe pain [7]. Similarly, Zaslansky et al., in a multicenter study involving approximately 6000 adult patients undergoing orthopedic and general surgeries across Europe and Israel, found that 70% and 48% of patients reported moderate and severe postoperative pain, respectively [8]. Evidence shows that over 75% of patients endure severe postoperative pain during recovery [4, 9, 10]. In Ethiopia, the incidence of moderate to severe postoperative pain has been documented to range from 47% to 100% [11]. When improperly managed, postoperative pain can have severe consequences, including compromised immune function, delayed wound healing, hypertension, blood clots, lung infections, and cardiovascular complications. It can also lead to psychological effects such as anxiety, depression, and insomnia [12]. Additionally, untreated pain may result in delayed recovery, increased mortality rates, reduced quality of life, and long-term opioid dependency [9]. Adequate knowledge and a positive attitude are essential for effective postoperative pain management (POPM) [13].

In Ethiopia, up to 91.4% of surgical patients experience untreated postoperative pain, with 88.2% reporting severe pain and 80.1% undertreated. This leads to delayed recovery, poor wound healing, and higher risks of complications like hypertension, pneumonia, and death. Psychological effects include anxiety, depression, and poor quality of life [14–16]. Over 74% of pediatric patients report moderate to severe pain, yet standardized assessment tools are rarely used. Despite available guidelines, the use of multimodal analgesia and patient-controlled techniques remains low due to cost, equipment shortages, and lack of training [17]. Critically, 72.1% of healthcare providers are unaware of national pain guidelines, and only 46.3% of patients receive proper pain scoring. This gap not only harms patients but also violates their right to adequate care [18, 19].

Pain management is a critical aspect of nursing care [20, 21]. International guidelines emphasize the importance of timely and effective pain relief to alleviate patient suffering [14]. Over the past 2 decades, the importance of managing postoperative pain effectively has been well recognized. Proper POPM can improve patient comfort, enhance mobility, accelerate recovery, shorten hospital stays, improve respiratory function, reduce cardiovascular strain, lower healthcare costs, and increase patient satisfaction [11, 22].

Nurses play a pivotal role in POPM by adhering to established protocols, accurately assessing pain levels, and creating individualized POPM plans. Enhancing nurses' understanding of pain is crucial to improving the quality of care. However, a gap often exists between nurses' perceptions of pain and patients' actual needs [23]. Studies highlight that limited knowledge, misconceptions about pain experiences, and inconsistent adherence to POPM guidelines hinder effective postoperative pain care [24–26]. Research indicates that pain remains undertreated due to several factors, with inadequate knowledge and negative attitudes among nurses being major contributors [27, 28]. POPM in Ethiopia is characterized by numerous systemic and professional challenges that compromise patient outcomes. In many healthcare settings, there is a lack of standardized pain assessment tools, inadequate access to effective analgesics, particularly opioids, and limited institutional guidelines to direct evidence-based POPM practices [29–31]. Nurses, who are the primary providers of bedside postoperative care, often have insufficient knowledge and training related to POPM, resulting in suboptimal assessment and intervention. Furthermore, sociocultural misconceptions about pain expression, fear of opioid addiction, and regulatory restrictions contribute to undertreatment of postoperative pain [32, 33]. These deficiencies not only hinder recovery and increase the risk of complications such as chronic pain but also diminish patient satisfaction and quality of care. Despite the central role nurses play in postoperative care, there remains a paucity of research assessing their knowledge, attitudes, and practices concerning POPM in the Ethiopian context. Investigating these factors is crucial for identifying educational gaps, informing policy reforms, and developing evidence-based interventions aimed at improving postoperative pain outcomes. The main research question aimed to be addressed by this study was the levels of knowledge, attitudes, and practices of nurses regarding POPM at Wolaita Sodo Comprehensive Specialized Hospital, and the factors which are associated with these outcomes.

Therefore, this study aimed at assessing the current levels of knowledge, attitudes, and practices of nurses regarding POPM and identifying the factors associated with these outcomes at Wolaita Sodo Comprehensive Specialized Hospital.

## 2. Methods

### 2.1. Design

This study utilized a cross-sectional design to assess nurses' knowledge, attitudes, and practices regarding POPM, as well as to identify factors influencing these dimensions. A validated questionnaire was used to collect quantifiable data on KAP and associated variables. This design was appropriate for examining relationships between variables using data collected at a single point.

### 2.2. Settings

The research was conducted at Wolaita Sodo Comprehensive Specialized Hospital, located in the Wolaita Sodo Zone, central southern Ethiopia. Data collection took place from September 6 to November 6, 2023.

### 2.3. Participants and Sample Size

The sample size was determined using the number of observations required per independent variable for regression analysis. Generally, the number of observations is 5–10 times the number of independent variables [34]. In this study, there were 10 independent variables with a significance level of 5% (*p* value = 0.05), an anticipated effect size of 0.15, and a statistical power of 0.8. Accounting for a 5% contingency for potential nonresponses, the calculated sample size was 124 participants.

**Figure 1:**
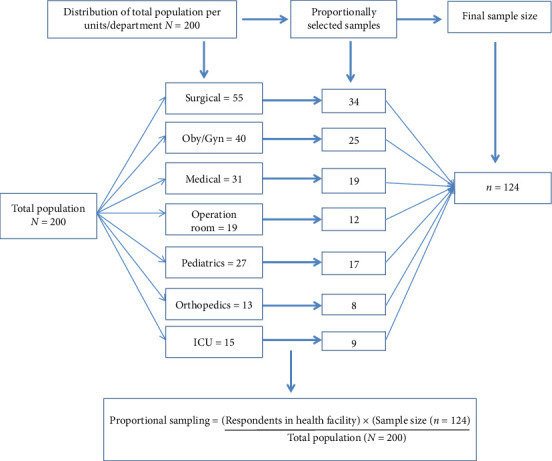
Study sampling frame.

After obtaining the list of nurses working in departments eligible for POPM, representative samples were calculated proportionally for each study area. The number of nurses selected from each department was determined based on the total number of nurses across all departments. Specifically, 34 nurses were from the surgical department, 25 nurses from obstetrics and gynecology, 19 nurses from the medical department, 17 nurses from pediatrics, 8 nurses from orthopedics, 9 nurses from the intensive care unit, and 12 nurses from the operating room. This proportional distribution ensured equitable representation of each department in accordance with their actual staffing levels. Then, study subjects were selected using a simple random sampling method. Nurses from the orthopedics ward, surgical ward, operating room, obstetrics and gynecology ward, intensive care unit, pediatrics and postanesthesia care unit, and those willing to consent were included to participate. Nurses who were severely ill or on maternity leave were excluded. See Figure 1.

### 2.4. Data Collection Instruments and Measurements

Data collection was carried out using a self-administered structured questionnaire divided into three sections: (1) demographic details, which included age, gender, marital status, years of service, educational level, work experience, in-service training, and personal pain experience; (2) questions related to knowledge and attitudes toward POPM; and (3) questions related to practice assessment concerning the performance of POPM.

### 2.5. Knowledge and Attitude

The researchers employed the Knowledge and Attitude Survey Regarding Pain (KASRP) to assess nurses' knowledge and attitudes regarding POPM. Developed by Ferrell and McGuire [35], this 36-item questionnaire is a widely recognized tool for evaluating healthcare professionals' understanding and perspectives on POPM. With permission from the original developers, a modified version of the KASRP, previously adapted in a study conducted in Ghana, was utilized in this study [13]. The instrument comprises 23 true/false items and 13 multiple-choice questions designed to evaluate nurses' knowledge and attitudes toward POPM. Specifically, 27 items assess knowledge, while the attitude section includes nine questions drawn from the same survey [13]. The internal consistency of the KASRP tools was confirmed with a reliability score of > 0.70 (Cronbach's alpha). Additionally, a panel of pain experts validated the content, referring to POPM guidelines provided by the American Pain Society, the World Health Organization, and the Agency for Health Care Policy and Research [13]. The cumulative correct responses, ranging from 0 to 36 points for knowledge and attitude items, were grouped into two categories based on the mean score. Scores below the mean were labeled as “inadequate knowledge,” while scores above the mean were classified as “adequate knowledge.” Likewise, attitude scores were categorized into “negative” for those below the mean and “positive” for those exceeding the median score.

### 2.6. Practice

The procedure for data analysis of the practice-related questions was similar to the knowledge section and assessed by using nurses' caring behavior regarding POPM tools developed by Erniyati [36]. The questionnaire included 36 items with two response options: “Yes” or “No.” To evaluate the action taken by nurses in managing postoperative pain, this questionnaire focuses on the alternative activities they perform or do not perform to help alleviate postoperative pain. A response was deemed correct if “Yes” was given for appropriate actions and the action was perceived as performed and “No” for inappropriate ones and the action was perceived as not performed, with each correct answer receiving one point. The coefficient reliability questionnaire was 0.78 [36].

To calculate the mean score for knowledge, attitude, and practice, each correct response is given a score of 1, while incorrect or unanswered questions receive a score of 0. The total score is the sum of all correct responses. This total is then converted to a percentage for easier interpretation and comparison, calculated by dividing the total score by the total number of questions and multiplying by 100. Then, the cumulative correct responses (i.e., ranging from 0 to 36 points) were divided into two categories based on the mean score. The results were categorized as “inadequate” for the scores below the mean line and “adequate” for those above the mean line.

### 2.7. Ethics Considerations

The study protocol was approved by the Medical Ethics Review Committees of Central South University (E2023177) and Wolaita Sodo University (WSU-IRRC/09/2023). Written informed consent was obtained from each participant before conducting the study.

### 2.8. Data Analysis

Data analysis was conducted using SPSS Version 28. Descriptive statistics was used to describe the frequency and percentage of study variables. Mean and standard deviation were used to describe continuous variables. An independent sample *T*-test was used for the variables with binary levels, such as gender, in-service training, marital status, work experience, and pain experience, to compare nurses' knowledge, attitude, and practice scores. One-way ANOVA was used for the variables of working services unit area, age in group, and level of education to compare nurse knowledge, attitude, and practice mean scores. The Pearson correlation coefficient (r) was utilized to examine the relationship between nurses' knowledge, attitudes, and practices. Multiple linear regressions were performed to determine associations of the study variable with nurses' POPM practices.

## 3. Results

### 3.1. Sociodemographic Characteristics of Respondents

A total of 124 respondents completed the questionnaire, achieving a 100% response rate. The surveys were distributed across various wards and units within the hospital. The participants' mean age was 31.7 years (SD = 6.2), with the majority being male (64.5%) and married (52.4%). Regarding education, 83.1% held bachelor's degrees, and 27.4% worked in the surgical unit. Additionally, 59.7% had not received in-service training, 58.9% had less than 3 years of work experience, and 86.3% reported having personal pain experiences. See Table 1 for details.

### 3.2. Knowledge Regarding POPM

The mean score for the 27 postoperative pain knowledge questions was 49.51 ± 9.51, with the percentage of correct responses for individual questions ranging between 30% and 70%. Based on the mean knowledge score, 54.8% of nurses in this study were classified as having an inadequate level of knowledge about POPM, as they scored below the mean. The majority of respondents have a poor understanding of opioid use safety as a medication used for surgical patients with substance abuse; 69.9% of nurses did not recognize and know opioids should be used in patients with a history of substance abuse. Furthermore, they have limited knowledge about opioid withdrawal symptoms; only 31.5% of participants know that following abrupt discontinuation of an opioid, physical dependence is manifested by sweating and yawning. Additionally, many respondents lack a comprehensive understanding of opioid dose conversion, a crucial aspect of safe prescription for POPM. For instance, 58.9% of participants are uncertain about the approximate equivalent of a 30 mg oral morphine dose to a 10 mg IV dose. However, many nurses know that opioid doses should be adjusted based on each patient's response and the best way to treat pain after surgery; 79% of nurses knew that after the first dose of an opioid analgesic was given, subsequent doses should be changed based on the patient's response, and 98% of participants knew that oral opioid analgesics are best for patients with persistent postoperative pain.  Table 2 presents a detailed summary of correct and incorrect responses to the postoperative pain knowledge inventory.

### 3.3. Attitude Toward POPM

The mean score for the nine attitude-related questions regarding pain was 43 ± 14.72. Based on this mean score, 59.7% (74 nurses) had a negative attitude toward POPM. Among the items, the most recognized were that 83.1% of nurses understood that patients' spiritual beliefs may lead them to think that pain and suffering are necessary, and 56.5% knew that patients may sleep despite experiencing severe pain. In contrast, the least known items were that 24.2% of nurses believed giving patients sterile water by injection (placebo) is a useful test to determine if the pain is real, and 26.6% thought patients should be encouraged to endure as much pain as possible before using an opioid. A detailed summary of the correct and wrong response rate for the postoperative pain attitude is presented in Table 3. See Table 3.

### 3.4. Practices Toward POPM

The mean score for the 36 practice-related questions on POPM was 71.05% (SD = 10.53), with the minimum and maximum scores being 56% and 94%, respectively. Based on the mean score for the 36 practice items, 38.7% of participants were classified as having good practice, while 61.3% exhibited poor practice regarding POPM.  Table 4 shows a detailed summary of the correct and incorrect response rates for the postoperative pain practice questions.

### 3.5. Respondents' Characteristics on Their Level of Knowledge, Attitude, and Practice Toward POPM

Independent *t*-tests and one-way ANOVA were used to assess whether a significant difference exists in the mean score of nurses according to their demographic characteristics. The practice was significantly associated with marital status, work experience, training on pain, and pain experience with an independent *t*-test showing a significant difference score among nurses who had work experience, *p* = 0.011; marital status, *p* = 0.003; received training in POPM, *p* < 0.001; and had pain experience, *p* < 0.001. Independent *t*-tests and one-way ANOVA did not show any significant association between attitude and sociodemographic characteristics of the respondents. In all cases, the *p* value was greater than 0.05. The gender and marital status of the participants were found to be significantly associated with knowledge (*p* < 0.05).  Table 5 summarizes the factors significantly associated with knowledge and practice.

### 3.6. Correlation Matrix Among Knowledge, Attitude, and Practices (*N* = 124)

The nurses' scores on their knowledge, attitudes, and practices were correlated using Pearson correlation. The correlation analysis using the Pearson coefficient revealed that there existed a moderate positive relation between practice and knowledge (*r* = 0.348, *p* value ≤0.001) and a weak positive correlation between attitude and practices (*r* = 0.247, *p* value = 0.006) of nurses toward POPM. There was no significant association between knowledge and attitude scores of the respondents. See Table 6.

### 3.7. Analysis of Factors Associated With POPM Practices

Linear regression analysis was used to investigate the impact of the factors that were found to have a significant association, using univariate analysis, with POPM practices. Overall, the regression model, as indicated by the R-square value of 0.228, explains approximately 22.8% of the variance in POPM practices. The R-square indicates that the independent factors in the model explain about 22.8% of the variation in POPM practices.

The variable marital status is associated with a coefficient of −4.175, a standard error of 1.724, and a beta value of −0.199. Marital status with a significance level of 0.017 was found to be a statistically significant predictor of POPM practices in this model. After adjusting for the variables included in the model, married respondents performed 4.175 times less practice than single respondents. Similarly, the variable training exhibits a coefficient of 4.672, a standard error of 1.817, and a beta value of 0.219. The model includes training as a statistically significant predictor of POPM practice, with a significance level of 0.011. The t-value of 2.571 and the significance level of 0.011 indicate that individuals who received in-service training are more likely to manage pain compared to those who did not receive training.

Similarly, the coefficient for pain experience is 6.124, with a standard error of 2.576 and a beta value of 0.201. The *t*-value of 2.377 and the significance level of 0.019 suggest that individuals who have experienced pain in their lifetime are more likely to practice POPM than those who have no pain experience. The variable work experience also shows statistically significant positive associations with POPM practices. The coefficient for work experience is 4.194, with a standard error of 1.725 and a beta value of 0.197. Work experience is included in the model as a statistically significant predictor of POPM practice, with a significance level of 0.017. The *t*-value of 2.432 and the significance level of 0.017 indicate that individuals who have more years of work experience are 4.194 more likely to manage pain compared to those who do not have work experience.  Table 7 presents a multiple linear regression model for POPM.

## 4. Discussion

This study evaluated the knowledge, attitudes, and practices of nurses toward POPM. Based on the mean knowledge score, 54.8% of nurses in this study have inadequate knowledge about POPM. This suggests that the respondents lack sufficient information on postoperative pain, highlighting a concern that deserves more attention. The findings from this study align with a recent systemic review and meta-analysis involving 7942 nurses which reported a mean score of 52.9% [37] and were lower than those of research conducted in Ghana 79.1% [13], and India 64.5% [38]. Insufficient coverage of pain topics in the Ethiopian undergraduate nursing curriculum, inadequate in-service training on postoperative pain, and restrictions on prescribing opioids secondary to fear of ethical implications as well as a misconception about the risk of opioid addiction could contribute to this outcome.

In terms of a thorough understanding of pain assessment and opioid medication, participants lack sufficient understanding of how to use the patient's words to estimate the severity of the patient's suffering as well as the fact that vital signs are not always accurate measures of a patient's pain intensity. This is notably lower than the finding from a study conducted in Italy at 95.5% [39], Jimma Medical Center at 58.8% [40]. Additionally, 29% of participants thought opioids should not be used during pain evaluation if the source of postoperative pain is unknown, which is again lower than findings at Jimma (54.5%) [40]. As well the participants in the study did not have enough information about the withdrawal symptoms that occur when opioid use is stopped. They also lacked knowledge about the dosage, duration, and onset of opioid medication as well as the replacement of different opioid medications. This finding aligns with other research results which indicate that opioid usage by nurses is the least known area [41, 42] and lower compared to Saudi nurses [43]. Furthermore, 45% of respondents believed that aspirin and other nonsteroidal anti-inflammatory drugs are ineffective for postoperative pain, and opioids should be avoided in patients with a history of substance abuse. This percentage is lower compared to nurses in Hawassa Hospital (75.4%) [44] and Italian nurses (66.8%) [39]. The reason for this could be due to the absence of POPM training, nurses are not allowed to prescribe opioid medications as part of their job, and they have limited access to new information due to their heavy workloads which can contribute to gaps in knowledge among nurses. Improve access to training and information resources by offering detailed, up-to-date training on both pharmacological and nonpharmacological POPM techniques. Explore the option of allowing nurses, especially advanced practice nurses like nurse practitioners, to prescribe opioid medications within their scope of practice and with proper oversight.

The nursing care behavior and practice toward POPM was poor, only 11.1% of respondents in this survey scored more than 80%, and only one participant scored above 90% among practice questions. Surprisingly, even though the majority of the respondents (83%) hold a bachelor's degree, their level of practice was less than 75%. The finding from this study is less than that of a study conducted in Bangladesh that reported a moderate degree of practice among nurses [45]. The disparity may stem from a knowledge gap, lack of POPM guidelines, unavailability of analgesics, and neglect of patients' pain and incorrect pain assessment. This is consistent with two studies reporting that the most common barrier to effective POPM is an incorrect assessment of pain by nurses. Ineffectiveness of pain relief measures and lack of knowledge on POPM and assessment [46, 47]highlight potential knowledge gaps and the absence of standardized guidelines specific to POPM, contributing to inconsistent care among nurses. To address these challenges, it is essential to develop an evidence-based guideline and training program. This program should emphasize the importance of timely pain assessment, correct analgesic administration, and monitor the effectiveness of pain relief.

The study finds that knowledge and practice scores were significantly associated with age and marital status, work experience, and training. This finding is consistent with other studies that reported a good correlation between POPM in nurses with more experience and those who trained [48, 49]. Nurses need to be highly educated, experienced, and well trained to provide higher quality care. This study finding showed a moderate positive correlation between the nurses' knowledge and their implementation of POPM practices. Nurses' attitudes were also found to have a positive correlation with their practices. There was no significant association between knowledge and attitude scores of the respondents. The finding aligns with a study conducted by Darjee, Dungpaeng, and Masingboon who revealed a positive correlation between nurses' knowledge, attitude, and practice regarding POPM [50]. Contrary to the findings of the present study, a study conducted in Nepal revealed that there is no statistically significant relationship among knowledge, attitude, and the practice score of the nurses regarding POPM [51].

## 5. Conclusion

This study identifies critical deficiencies in nurses' knowledge, attitudes, and practices regarding POPM, with inadequate training and poor assessment contributing to suboptimal patient outcomes. A positive correlation between knowledge and practice emphasizes the need for structured education, while the weak link between attitude and practice suggests that attitude alone is insufficient without adequate support. To address these gaps, nursing curricula should incorporate evidence-based POPM content, and hospitals like WSCSH should implement regular, technology-enhanced in-service training. The Ministry of Health must enforce continuing professional development through standardized training and audits. A coordinated effort among policymakers, educators, and healthcare administrators is essential to improve clinical practice and patient outcomes.

### 5.1. Recommendation

To improve nurses' knowledge and practice in POPM, WSCSH should implement regular, evidence-based in-service training focused on pain assessment, pharmacologic safety, and multimodal strategies. A blended learning model incorporating workshops and digital tools, including AI-powered simulations, can enhance accessibility and engagement. International frameworks such as the WHO Pain Ladder, the Pain Resource Nurse (PRN) model, and ASPMN modules should be contextually adapted to align with local language, resources, and cultural practices. Nursing curricula should be updated to include essential content on pain physiology, assessment, and management, ensuring alignment between academic instruction and clinical practice. The Ministry of Health should mandate CPD in POPM through standardized training and periodic audits to ensure sustained competency. Collaboration with educational institutions can support ongoing professional development and research. Integrating AI technologies into nursing education and decision-making may further support evidence-based practice. Finally, multicenter, longitudinal studies are recommended to evaluate context-specific interventions and the impact of technology-enhanced education on clinical outcomes.

### 5.2. Limitation of the Study

One key limitation of this study is the reliance on self-reported data to assess nurses' practices in POPM. Self-reporting may introduce biases, such as social desirability bias, where participants provide responses they perceive as favorable rather than reflecting their actual practices. This discrepancy could affect the accuracy of the findings. Additionally, the study's sample was relatively small and drawn exclusively from a single institution (WSUSCH), which may limit the generalizability of the results to nurses in other hospitals or regions. To strengthen external validity, future research should include larger, more diverse samples from multiple healthcare settings.

## Figures and Tables

**Table 1:** Sociodemographic characteristics of the study participants (124).

Variables	Categories	Frequency	Percent (%)
Sex	Male	80	64.5
	Female	44	35.5

Age	23–29	49	39.5
	30–39	60	48.4
	40 and above	15	12.1

**Mean age of participants = 31.7 years, SD = 6.2**			

Marital status	Single	59	47.6
	Married	65	52.4

Working service area	Surgical	34	27.4
	Oby/Gyn	25	20.2
	Medical	19	15.3
	Pediatrics	17	13.7
	Orthopedics	8	6.5
	ICU	9	7.3
	Operation room	12	9.7

Work experience in years	< 3 years	73	58.9
	≥ 5 years	51	41.1

**Mean = 5.86 years, SD = 6.06**			

Level of education	Diploma	9	7.3
	Degree	103	83.1
	Master	12	9.7

Training on POPM	Yes	50	40.3
	No	74	59.7

Pain experience	Yes	107	86.3
	No	17	13.7

**Table 2:** Nurses' knowledge of postoperative pain management by item (*n* = 124).

No.	Items	Correct *N* (%)	Wrong *N* (%)
1	Vital signs are always reliable indicators of the intensity of a patient's pain.	36 (29.0%)	88 (71.0%)
2	Because their nervous system is underdeveloped, children < 2 years of age have decreased pain sensitivity and limited memory of painful experiences.	44 (35.5%)	80 (64.5%)
3	Aspirin and other nonsteroidal anti-inflammatory agents are not effective analgesics for acute postoperative pain.	56 (45.2%)	68 (54.8%)
4	Respiratory depression rarely occurs in patients who have been receiving stable doses of opioids over a period of months.	87 (70.2%)	37 (29.8%)
5	Combining analgesics that work by different mechanisms may result in better pain control with fewer side effects than using a single analgesic agent.	90 (72.6%)	34 (27.4%)
6	The usual duration of analgesia of 1–2 mg morphine IV is 4–5 h.	44 (35.5%)	80 (64.5%)
7	Opioids should not be used in patients with a history of substance abuse.	41 (33.1%)	83 (66.9%)
8	After an initial dose of opioid analgesic is given, subsequent doses should be adjusted in accordance with the individual patient's response.	98 (79%)	26 (21.0%)
9	If the source of the patient's pain is unknown, opioids should not be used during pain evaluation, as this could mask the ability to correctly diagnose the cause of pain.	36 (29.0%)	88 (71.0%)
10	Anticonvulsant drugs such as gabapentin (Neurontin) produce optimal pain relief after a single dose.	49 (39.5%)	75 (60.5%)
11	Benzodiazepines are not effective pain relievers and are rarely recommended as part of an analgesic regiment.	72 (58.1%)	52 (41.9%)
12	Narcotic/opioid addiction is defined as a chronic neurobiological disease, characterized by behaviors of impaired control over drug use, compulsive use, continued use despite harm, and craving.	65 (52.4%)	59 (47.6%)
13	The term “equianalgesia” means approximately equal analgesia.	90 (72.6%)	34 (27.4%)
14	Sedation assessment is recommended during opioid pain management.	79 (63.7%)	45 (36.3%)
15	Pethidine 75 mg IM is approximately equal to morphine 10 mg IM.	48 (38.7%)	76 (61.3%)
16	Vicodin (hydrocodone 5 mg + acetaminophen 300 mg) PO is approximately equal to 5–10 mg of morphine PO.	59 (47.6%)	65 (52.4%)
17	The recommended route of administration of opioid analgesics for patients with persistent postoperative pain is oral.	121 (97.6%)	3 (2.4%)
18	The recommended route of administration of opioid analgesics for patients with brief, severe postoperative pain is IV.	65 (52.4%)	59 (47.6%)
19	A 30 mg dose of oral morphine is approximately equivalent to 10 mg IV.	73 (58.9%)	51 (41.1%)
20	Analgesics for postoperative pain should initially be given. (Around the clock on a fixed schedule).	66 (53.2%)	58 (46.8%)
21	The most accurate judge of the intensity of patient's pain is. (The patient).	33 (26.6%)	91 (73.4%)
22	The likelihood that patients who develop pain already have an alcohol and/or drug abuse problem is 15%.	47 (37.9%)	77 (62.1%)
23	The time to peak effect for morphine given IV is 15 min.	69 (55.6%)	55 (44.4%)
24	The time to peak effect for morphine given orally is 1–2 h.	59 (47.6%)	65 (52.4%)
25	Following abrupt discontinuation of an opioid, physical dependence is manifested by sweating and yawning.	39 (31.5%)	85 (68.5%)
26	Which statement is true regarding opioid induced respiratory depression? (Obstructive sleep apnea is an important risk factor).	54 (43.5%)	70 (56.5%)
27	The following nondrug methods are useful for combining with treatment of postoperative pain (mediation, reading, deep breathing, and cool/heat).	75 (60.5%)	49 (39.5%)

**Table 3:** Nurses' attitude of postoperative pain management by items (*n* = 124).

No.	Items	Correct *N* (%)	Wrong *N* (%)
1	Patients who can be distracted from pain usually do not have severe pain.	41 (33.1%)	83 (66.9%)
2	Patients may sleep in spite of severe pain.	70 (56.5%)	54 (43.5%)
3	Elderly patients cannot tolerate opioids for pain relief.	40 (32.3%)	84 (67.7%)
4	Patients should be encouraged to endure as much pain as possible before using an opioid.	33 (26.6%)	91 (73.4%)
5	Children less than 11 years old cannot reliably report pain, so clinicians should rely solely on the parent's assessment of the child's pain intensity.	37 (29.8%)	87 (70.2%)
6	Patients' spiritual beliefs may lead them to think that pain and suffering are necessary.	103 (83.1%)	21 (16.9%)
7	Giving patients sterile water by injection (placebo) is a useful test to determine if the pain is real.	30 (24.2%)	94 (75.8%)
8	The most likely reason a patient with pain would request increased doses of pain medication is the patient is experiencing increased pain.	67 (54.0%)	57 (46.0%)
9	The best approach for cultural considerations in caring patients in pain should be individually assessed to determine cultural influence.	48 (38.7%)	76 (61.3)

**Table 4:** Nurses' practice of postoperative pain management by item (*n* = 124).

No.	Item	Correct answer	Correct *N* (%)	Wrong *N* (%)
1	Use observation to determine pain.	Yes	117 (94.4%)	7 (5.6%)
2	Ask patients to determine pain.	Yes	108 (87.1%)	16 (12.9%)
3	Use the pain scale to describe pain intensity.	Yes	106 (85.5%)	18 (14.5%)
4	Ask patients to evaluate their pain after surgery	Yes	92 (74.2%)	32 (25.8%)
5	Ask about frequency of their pain experience.	Yes	89 (71.8%)	35 (28.2%)
6	Ask to describe the pain by own words.	Yes	94 (75.8%)	30 (24.2%)
7	Ask to locate the area of pain.	Yes	89 (71.8%)	35 (28.2%)
8	Ask about the most severe pain after surgery.	Yes	97 (78.2%)	27 (21.8%)
9	Ask about the least severe pain after surgery.	Yes	96 (77.4%)	28 (22.6%)
10	Ask about the average pain after surgery.	Yes	96 (77.4%)	28 (22.6%)
11	Ask about the presence of any other symptoms.	Yes	97 (78.2%)	27 (21.8%)
12	Ask about the intensity of pain before giving pain killers.	Yes	91 (73.4%)	33 (26.6%)
13	Ask about the intensity of pain after giving pain drug.	Yes	90 (72.6%)	34 (27.4%)
14	Ask about the factors that increase the intensity of pain.	Yes	85 (68.5%)	39 (31.5%)
15	Ask about the factors that reduce the intensity of pain.	Yes	89 (71.8%)	35 (28.2%)
16	Ask about the cause if their pain becomes worst.	Yes	87 (70.2%)	37 (29.8%)
17	Ask about nonpharmacological method to reduce pain.	Yes	84 (67.7%)	40 (32.3%)
18	Ask about the side effects of pain medication.	Yes	82 (66.1%)	42 (33.9%)
19	Give prescribed pain medication on a fixed schedule.	Yes	92 (74.2%)	32 (25.8%)
20	Give medication as necessary.	Yes	80 (64.5%)	44 (35.5%
21	Explain the pain experience after surgery.	Yes	85 (68.5%)	39 (31.5%)
22	Teach alternative methods to reduce pain.	Yes	92 (74.2%)	32 (25.8%)
23	Explain side effects of pain medication.	Yes	74 (59.7%)	50 (40.3%)
24	Suggest and explain drug addiction to reduce fear.	Yes	66 (53.2%)	58 (46.8%)
25	Teach the importance of pain evaluation	Yes	67 (54.0%)	57 (46.0%)
26	Provide comfort after surgery.	Yes	82 (66.1%)	42 (33.9%)
27	Help position comfortably after surgery.	Yes	76 (61.3%)	48 (38.7%)
28	Help patients when they need help.	Yes	80 (64.5%)	44 (35.5%)
29	Help have enough sleep.	Yes	80 (64.5%)	44 (35.5%)
30	Spend time to reduce pain after surgery.	Yes	72 (58.1%)	52 (41.9%)
31	Teach to support their surgical wound.	Yes	79 (63.7%)	45 (36.3%)
32	Help support pain area.	Yes	101 (81.5%)	23 (18.5%)
33	Take care of patients' wounds.	Yes	85 (68.5%)	39 (31.5%)
34	Provide alternative activities to alleviate pain.	Yes	85 (68.5%)	39 (31.5%)
35	Teach patients to perform distracted activities.	Yes	88 (71.0%)	36 (29.0%)
36	Help ambulate such as sitting up.	Yes	98 (79.0%)	26 (21.0%)

**Table 5:** Summary of factors significantly associated with knowledge and practice.

Variable	Category	*N*	%	Mean	SD	*t*-value	(df)	*p* value
*Knowledge*								
Sex	M	80	64.5	51.41	9.50	3.11	122	0.002^∗∗^
	F	44	35.5	46.05	8.59			
Marital status	Single	59	47.6	51.81	10.32	2.63	122	0.010^∗^
	Married	65	52.4	47.42	8.12			

*Practice*								
Marital status	Single	59	47.6	73.96	10.97	3.01	122	0.003^∗∗^
	Married	65	52.4	68.41	9.45			
Work experience	< 3 years	70	58.9	69.05	10.58	−2.59	122	0.011^∗^
	≥ 5 years	54	13.7	73.92	9.98			
Training on pain	Yes	51	41.1	75.08	10.97	−3.74	122	< 0.001^∗∗^
	No	73	58.9	68.24	9.30			
Pain experience	Yes	107	86.3	72.31	10.54	−3.49	122	< 0.001^∗∗^
	No	17	13.7	63.12	6.28			

^∗^Respondents' characteristics are significant at the 0.05 level.

^∗∗^Respondents' characteristics are significant at the 0.01 level.

**Table 6:** Pearson correlation among knowledge, attitude, and practice.

Variables	Knowledge	Attitude	Practice
Knowledge			
Attitude	0.168		
Practice	0.348^∗∗^	0.247^∗^	

^∗^Correlation is significant at the 0.05 level (2-tailed).

^∗∗^Correlation is significant at the 0.01 level (2-tailed).

**Table 7:** Multiple linear regression model for postoperative pain management.

	Unstandardized coefficients		Standardized coefficients	*T*-value	*p* value
	B	Std. error	Beta		
(Constant)	64.311	2.603		24.708	< 0.001
Training	4.672	1.817	0.219	2.571	0.011
Pain experience	6.124	2.576	0.201	2.377	0.019
Work experience	4.194	1.725	0.197	2.432	0.017
Marital status	−4.175	1.724	−0.199	−2.421	0.017

*Note:* Dependent variable: postoperative pain management practice, R-square 0.228, *F* = 8.79, *p* ≤ 0.001.

## Data Availability

The data that support the findings of this study are available from the corresponding author upon reasonable request.
